# Live stream marketing and consumers’ purchase intention: An IT affordance perspective using the S-O-R paradigm

**DOI:** 10.3389/fpsyg.2023.1069050

**Published:** 2023-02-01

**Authors:** Lu Zhang, Min Chen, Ahmad M. A. Zamil

**Affiliations:** ^1^School of Journalism and Communication, Shanghai University, Shanghai, China; ^2^School of Business, Fuyang Normal University, Fuyang, China; ^3^School of Communication, Soochow University, Suzhou, Jiangsu, China; ^4^Department of Marketing, College of Business Administration, Prince Sattam Bin Abdulaziz University, Al-Kharj, Saudi Arabia

**Keywords:** live marketing, social commerce, IT affordance, SOR, purchase intention

## Abstract

Recent years have seen a shift in the online retail industry toward a greater emphasis on live marketing. The growth of social media commerce on the internet demonstrates the popularity of Livestream shopping. Although there has been a rise in interest in studying live streaming, a comprehensive model describing why consumers are willing to consistently employ this novel sales format has yet to be developed. Hence, the present study develops a model to determine the factors influencing consumers’ live-stream shopping intention by employing the affordance lens and S-O-R model. The online data was collected through the Wenjuanxing website from the users of live-streaming platforms such as Taobao.com, Mogujie.com, and JD.com. Results showed that (*N* = 434): trust can be enhanced through visibility, metavoicing, trading affordance, guidance shopping and interactivity that consequently affects consumer purchase intention. In addition, this study highlights the theoretical and managerial implications for social commerce.

## 1. Introduction

Online social networking sites have significantly altered how users interact with others, share personal information, and go about their daily lives (Hu et al., 2015; Chao et al., 2022). Online social networking services employ a variety of features, such as live streaming, instant messaging, fan pages, and chats, to foster social connections among their members. Facebook Live, Instagram Stories, and YouTube are just a few examples of the live-streaming platforms available (Chao et al., 2022). Live streaming, an internet-based multimedia entertainment, has quickly acquired popularity worldwide, particularly in highly interactive platforms/products like online gaming, travel, and shopping (Tong et al., 2022). Recently, as a result of COVID-19, consumers have favored live shopping with contact-free service and instant involvement over high-risk retail shopping. Unlike the more typical teleshopping format, which is centered only on a television set, live shopping is a more interactive and social experience (Yen, 2020; Tong et al., 2022).

Teleshopping may optimize product display *via* engaging TV presenter explanations, in-depth demonstrations, and live models. On the other hand, teleshopping is a one-way communication that lacks timely and obvious engagement with customers (Yen, 2020). Live shopping enables two-way conversation with customers in real-time, may be used in more situations than teleshopping and is compatible with many electronic devices (including smartphones and PCs; Tong et al., 2022). Live shopping is a new alternative to traditional online shopping that deserves attention.

Many aspects of conventional social commerce have been altered by live streaming. For example, while purchasing online, shoppers can read descriptions and look at photographs (Zhang et al., 2022). Live streaming shopping, on the other hand, enables streamers (online vendors) to present products in real-time videos, providing buyers with more in-depth information about the products on offer (Wongkitrungrueng and Assarut, 2018). Consumers may ask questions *via* the bullet screen, and retailers can respond by utilizing real-time live streaming to provide highly tailored services and recommendations to customers, which can significantly affect their purchasing decisions (Wongkitrungrueng and Assarut, 2018; Sun et al., 2019). Second, in conventional social commerce, buyers who have inquiries about a particular product must move off that page before getting in touch with the seller. Comparatively, during a live-streamed shopping experience, consumers may interact with streamers by typing inquiries onto a bulletin board and receiving immediate answers (Sun et al., 2019). As a third point, in conventional social commerce, vendors cannot advise buyers. The lack of facial interaction between buyer and seller heightens the sense of danger associated with purchasing online. It is obvious that live-streaming online shopping is an excellent solution to this problem.

Businesses and entrepreneurs have taken an interest in live streaming due to its lucrative benefits (Apasrawirote and Yawised, 2022). Consequently, companies through live-streaming platforms saw explosive growth in emerging markets like China. IResearch’s 2021 report on Chinese live-streaming commerce states that live-streaming commerce in China is expected to rise from its current size of over 1.2 trillion RMB in 2020 to over 4.9 trillion RMB by 2023 (Ma et al., 2022). The live-streaming business is worthy of further study, considering its substantial potential financial impact (Xie et al., 2022).

This study’s primary audience was local Chinese customers who like live-streamed shopping for two reasons. According to the Digital 2020 Global Overview study, 75% of the younger generation uses the internet for informational purposes and retail buying (Apasrawirote and Yawised, 2022). China has seen tremendous growth in live streaming shopping behavior recently, it is found that live streaming market has reached to RMB2.27 trillion in 2021 which is expected to be RMB 4.9 trillion by 2023 (Statista, 2021). This suggested that dominant factors had a role in determining live shopping behavior. Therefore, it would be helpful to investigate the variables affecting customers’ propensity to buy things during live broadcasting. In this regard, trust is one of the main factors in lie streaming. In social commerce, trust is a very crucial element of consumer behavior. It is sometimes tough for a buyer to trust the seller because they can provide mendacious information (Dong and Wang, 2018). In addition, they can suffer from negative transaction experiences by getting fake products, low-quality products and wrong information.

Interestingly, live streaming allows the consumer to watch actual products and sellers, which develops consumers’ trust in the seller and product, which was difficult in conventional social commerce. However, research has also said that live streaming is a human-computer interaction requiring human psychology and IT features. The concept of affordance enables us to consider the technical aspects of live-streaming commerce and the customers’ perceptions of these aspects (Leonardi et al., 2013; Parchoma, 2014; Treem and Leonardi, 2016). According to Volkoff and Strong (2013), affordance refers to the likelihood that an object influences an individual to perform a particular activity. The connection between users and technical features produces affordance (Dong and Wang, 2018). It is found that affordance is created when people use and interact with live-streaming shopping (Aladwani, 2017). Affordability has been utilized in several past studies on social trade (Dong and Wang, 2018; Lin et al., 2019).

In addition, although there are more and more studies on live-streaming consumer behavior, there is still not a comprehensive theorization of the factors that impact live-streaming shopping behavior, especially after the COVID-19 pandemic lockdowns, when a lot of companies started to use live streaming for its advantages. Moreover, Academic research on IT affordance as stimuli for live streaming is rare (Sun et al., 2019), despite its expanding trend in consumer behavior. Keeping this in mind, the stimulus organism response (SOR) model was chosen as the primary theory for studying factors affecting live streaming buying behavior. Because the SOR model provides researchers with a sequential mechanism of complex phenomena of human behavior (Jabeen et al., 2022).

All of these suggest that examining live-streaming shopping behavior would provide a valuable context for such an initiative, theoretically and practically. The current study contributes in the following way. First, the present research integrates IT affordance and the SOR model to better understand how consumers’ purchase intention develops. Second, the current study takes multidimensional trust (trust in the seller and trust in products) as an organism of consumer behavior that leads toward the purchase intention. Third, the current study is finding how consumers’ trust can be built in live streaming shopping behavior through IT affordance, which is obsolete in the literature.

The rest of the paper is as follows: section 2 consists of the literature review and hypotheses development. Section 3 consists of the research methodology. Section 4 provides all information regarding research analysis and results, while section 5 discusses the reasons for the results and the theoretical and practical contribution of the study.

## 2. Literature review and theoretical background

### 2.1. Live streaming in social commerce

Social commerce, a type of e-commerce, requires using social media platforms that facilitate communication and engagement to facilitate and improve online transactions and retail experiences (Shen and Eder, 2011; Liang and Turban, 2014). Live-streaming commerce is a relatively new business model that combines traditional live broadcasting, social trading, and online buying and selling, made possible by the proliferation of mobile communication technologies in recent years (Todd and Melancon, 2018). Live streaming commerce greatly enhances interactivity (Xue et al., 2020; Kang et al., 2021) because live streaming is enabled by web 3.0 technology which enables real-time multidimensional e-commerce. However, Web 1.0 technology allows one-to-one e-commerce and Web 2.0 technology is limited to many-to-many e-commerce (Mou and Benyoucef, 2021).

Live-streaming commerce is the latest social commerce that includes high human-computer interaction. This technology uses one or more communication systems that can instantly transfer videos and audio to other devices, locations and areas that allow users to experience presence (Chen and Lin, 2018). For instance, Cheung and Huang (2011) focused on live streaming on e-sports, while Sjöblom and Hamari (2017) researched the motivation behind watching live video games. A recent study has provided novel insights into live-streaming commerce, which explains consumer engagement through different values, i.e., utilitarian, hedonic and symbolic values (Wongkitrungrueng and Assarut, 2018). In contrast, other researchers have discussed the users’ intrinsic and extrinsic motivation perspectives, which affect streamers’ intention to broadcast live streaming (Zhao et al., 2018). At the same time, some other researchers have examined the effect of consumer perception and design aspects of live streaming on consumers’ intentions (Ho and Yang, 2015; Chen and Lin, 2018).

Customers love the option to purchase through live-streaming shopping since it is simple to inspect things from different angles and ask pertinent questions (Lu et al., 2018). However, live streaming involves significant human-computer interaction, and it is vital to examine the technical characteristics and the customers’ perceptions of those characteristics. However, few studies have investigated the impact of live-streaming shopping on consumers’ purchase intentions. In order to examine customer purchase intention, the current research thoroughly investigates both the technical characteristics and the customers’ views in the context of live-streaming commerce. In this regard, a theoretical foundation has been taken by the stimulus organism model (SOR model) integrated with affordance theory to understand the phenomena better.

### 2.2. The SOR model

The stimulus organism and response (S-O-R) theory presented by Mehrabian and Russell (1974) is an approach to environmental psychology. The SOR model has three stages, i.e., environmental or external stimuli, consumers’ internal state or organism and response. First, stimuli affect consumers’ organism, which ultimately influences consumers’ responses. Previous researchers in the online context characterized stimuli as environmental or external elements, including website features and social aspects (Zhang et al., 2014). Internal states include users’ cognitive and emotional states, such as perceptions, experiences, and evaluations (Friedrich et al., 2019).

Individual behavior is represented by the responses, which include active and passive participation behaviors, which can be physical or online communications (Xue et al., 2020). The SOR model has been extensively used by many e-commerce, and s-commerce researchers, such as Friedrich et al. (2019) study about website stickiness, Yuan et al. (2020) study about customer loyalty Islam and Rahman (2017) research about online community and Hu et al. (2017) conducted research on live streaming. The current study adopted the SOR model due to two main reasons. First, The SOR model is extensively accepted in marketing and consumer studies and is widely used by online shopping researchers, as mentioned above. Hence, it can be beneficial in assessing consumers’ purchase intention, particularly in live streaming. Second, Live streaming commerce is very distinct from conventional e-commerce because it is based on social interactions between streamers and users and between users themselves. The S-O-R model lets us consider these unique aspects of live-streaming shopping behavior and build a vigorous and structured model for developing consumer responses initiated from live interactions as an external stimulus on users’ experiences. Internal states ultimately lead toward consumer responses.

The current study used the SOR model to develop a comprehensive model of consumers’ purchase intention from live streaming. In this regard, the present study used IT affordance (Guidance shopping, Meta voicing, visibility, social connecting and triggered attendance) as an external stimulus (S) based on IT affordance (Dong and Wang, 2018). These IT affordances develop trust in products and sellers, which refers to the consumers’ internal state (O) (Wongkitrungrueng and Assarut, 2018). This organism leads toward consumer response which is the purchase intention by the consumer through online live streaming (Sun et al., 2019).

### 2.3. Live streaming and affordance theory

Affordance can be defined as “the potential for behaviors associated with achieving an immediate concrete outcome and arising from the relation between an object (e.g., an IT artifact) and a goal-oriented actor or actors” (Bygstad et al., 2016, p. 87). Several fields of study have distinct affordance definitions. According to Volkoff and Strong (2013), affordance denotes the likelihood that an object influences an individual to perform a particular activity. Affordance has been extensively investigated in the fields of IS (Leonardi, 2011; Piccoli, 2017) and social media (Argyris and Monu, 2015).

Similarly, in the live-streaming context, when customers watch live streamers for shopping or product information, they discover and make opinions regarding live-streaming shopping features. Thus, it can be said that affordance permits to study of users’ perceptions and technical aspects simultaneously rather than separately (Leonardi et al., 2013; Parchoma, 2014). Previous research identified affordance features in various ways, demonstrating that affordance features can differ depending on the content (Koroleva and Kane, 2017), e.g., editability, persistence, visibility, and association (Treem and Leonardi, 2016; Chen et al., 2020); social contacting affordance, visibility affordance, triggered attending affordance, metavoicing affordance, shopping guidance affordance, and trading (Argyris and Monu, 2015); shopping guidance, visibility, triggered attending, social contracting, metavoicing, and trading (Dong and Wang, 2018); stickiness, word of mouth, and interactivity (Lin et al., 2019); visibility, metavoicing, and shopping guidance (Sun et al., 2019; Tuncer, 2021); and information, interactivity, and navigation (Shao et al., 2020).

According to a review of the literature, affordance theory has been widely used in the context of numerous research to investigate the technological artifact’s effect on individuals’ perceptions and behavioral responses, especially in recent years (Dong and Wang, 2018; Sun et al., 2019; Shao et al., 2020; Fang et al., 2021). According to the relevant research, social commerce affordance has a variety of effects, including social ties (Dong and Wang, 2018), customer engagement (Sun et al., 2019), relationship quality and brand experience (Fang et al., 2021) and social commerce (Tuncer, 2021). According to the data in this research, it can influence an individual’s cognitive and affective reactions regarding affordances and environmental variables. As a result, this study illustrates how the live streaming IT affordance affects the customer’s behavioral intention through trust.

### 2.4. Guidance shopping

Guidance shopping can be defined as offering products and services tailored to customers’ needs, interests and demand (Dong and Wang, 2018). Guidance shopping affordance can be helpful for buyers by offering personalized services. This is an interactive process based on an interaction in which vendors learn about the customers’ interests. The advice provided by streamers in live streaming purchasing is based on the consumers’ unique needs. Customers will therefore concentrate their attention toward streamers during live-streaming, which will assist them in creating immersion (Yim et al., 2017). Trust happens when users are immersed in a seamless process that addresses their intrinsic utilitarian incentives and requirements (Fang et al., 2018).

Meanwhile, guidance shopping affordance enhances the perceived utilitarian value when assisting clients in resolving problems using live-streaming shopping (Dong and Wang, 2018), ultimately improving their trust in the product (Wongkitrungrueng and Assarut, 2018). Clients can approach streamers directly for purchasing assistance, while sellers can deliver product information to users depending on their particular needs. Social media networks may respond to consumers’ tastes and demands with a personalized product or service architecture. This raises the possibility that the consumer will believe that the platform understands their preferences and requirements (Zhang and Curley, 2017). It also suggests that the vendor is responsible and trustworthy (Xiao and Benbasat, 2011).

Furthermore, guiding shopping affordance can boost customer-streamer contact (Dong and Wang, 2018). Consumers feel the information and recommendations by the seller are accurate, and they can find the best-fit product for themselves (Wongkitrungrueng and Assarut, 2018). In this complete process, customers get their trust in the buyer and the product because they physically watch both of them. As a result, we hypothesize:

*H1*: Guidance shopping is positively and significantly associated with trust.

### 2.5. Meta voicing affordance

Metavoicing affordance refers to meeting buyers’ needs in terms of finding useful information concerning products and services during interaction between the buyer and seller (Dong et al., 2016). The metavoicing feature allows sellers and customers to rate one another and give product reviews or feedback during transactions (Sun et al., 2019). When customers share their thoughts on Live streaming, they engage with streamers in an informal interactive channel, allowing the sharing of product and service-related details and assisting in the resolution of transaction-related problems. Metavoicing affordance increases buyer–seller involvement by gathering individual voices and comments into an interactive discussion (Dong et al., 2016). Customers with product-related questions can pose them straight to streamers through live chat rooms (Chen and Lin, 2018; Fang et al., 2018). Customers can ask follow-up questions by replying to streamers’ responses, and this process continues until the resolution of consumer queries.

Therefore, metavoicing affordance promotes direct dialog between streamers and customers, creating an excellent impression among consumers and reducing the perceived gap between streamers and consumers (Lv et al., 2018), which build a sense of trust. This enables consumers to concentrate on live shopping activities, creating a sense of immersion and presence (Sun et al., 2019). Moreover, buyers have the power to express their opinions by writing comments and ratings on social commerce or live streaming chat rooms, which increase valuable information on live streaming sessions through metavoicing. Other buyers consider this information a trustworthy source on social media (Benlian et al., 2014), which can increase the consumer’s trust. However, a recent study on social commerce intention could not find any association between metavoicing and trust. So this relationship requires further clarification.

*H2*: Metavoicing is positively and significantly associated with trust.

### 2.6. Visibility

Visibility affordance refers to the provision of simple access to products and visibility of product information in relation to consumers’ social media shopping behavior (Dong et al., 2016). Visibility affordance decreases product ambiguity and perceived risk of the consumers by visualization of live products, their images and information. In this regard, sellers can concurrently display product images and relevant information. Customers are more receptive to interactions with sellers who exhibit accurate knowledge. Moreover, internet buyers rely heavily on text descriptions and product photographs (Ma et al., 2022). In this regard, Live streaming can instantly transmit visuals and audio from one area to another (Chen and Lin, 2018). The visual cues could increase interaction transparency, decreasing the negative spillover impact resulting from information asymmetry and product uncertainty (Sun et al., 2019).

Live-streaming shopping uses web video technologies, making it a prominent product promotion platform. Customers usually pay attention to live streamings to get product information, knowledge and live experiences for decision-making, which develops consumers’ trust in the product (Wongkitrungrueng and Assarut, 2018). Furthermore, the vividness of live-streaming purchasing makes it easier to attract clients.

On the other side, it can be used to broadcast detailed information for the buyers, and sellers can also teach consumers how to use this particular product through live streaming sessions. As a result, users can learn about actual products and watch sellers/streamers as “actual individuals” (Li, 2019), reducing the risk of ordering from an unknown entity. Furthermore, Wongkitrungrueng and Assarut (2018) found that product or seller visibility increases consumers’ utilitarian value and builds the consumer’s trust in the seller and the product through available information.

*H3*: Visibility affordance is positively and significantly associated with trust.

### 2.7. Trading affordance

Trading affordance refers to making transaction systems easy for the customers during shopping by offering different payment options (Dong and Wang, 2018). Trading affordance facilitates deals by giving purchasers a variety of payment choices. When a buyer pays their bill, the transaction is complete. When the transaction is over, buyers and sellers usually want to communicate more (Dong and Wang, 2018). Consumers hope to receive more benefits or a discount on their future purchases. Sellers wish to convert this one-time or single transaction into a recurring purchase. They also expect purchasers to leave excellent reviews and share product information on social media. As a result, trading affordance can improve buyer–seller interaction even further. Moreover, live streaming help consumers ask instant questions regarding products (Kang et al., 2021) and different payment options and trading facilities from the seller side. So the transaction process can occur smoothly as sellers on social media are not the big giants but small sellers who sell their products by explaining their features, customer needs and fare prices (Wongkitrungrueng and Assarut, 2018).

With the advancement of technology, consumers can pay online through cashless payment systems such as Ali pay, WeChat pay, and e-banking (Miao et al., 2022). Trading affordance enables consumers to identify the person to whom and for what they are paying, reducing the risk of financial fraud on social media and increasing trust. A recent study found that trading affordance facilitates donors to pay on charitable crowdfunding through social media platforms. Because the smooth systems and effective payment methods build trust in the receiver (Jiao et al., 2021), based on the above discussion, trading affordance build trust in the seller. Hence it can be hypothesized that.

*H4*: Trading affordance is positively and significantly associated with trust.

### 2.8. Interactivity

Interactivity refers to the degree and depth of the interaction that happens during two parties’ reciprocal communication (Ma et al., 2022). Live-streaming shopping is a type of s-commerce mainly recognized for interaction. In the live-streaming shopping experience, users can interact with sellers and other users or consumers in real-time. Interactivity refers to the degree and richness of interaction between two-party conversations (Kang et al., 2021). Live stream commerce enables sellers to increase their responsiveness regarding customer queries and personalized information. Because in live chats, sellers or streamers can respond to their personal questions quickly compared to traditional methods, which increases interactivity (Xue et al., 2020), clears their doubts about the products and increase consumers’ trust. As Wang et al. (2019) said, users in live chat rooms can use marketing message information to identify different product features, functions, usage methods and purchasing products.

Similarly, along with personalized information, live streamers can quickly respond to the viewers’ requests or queries, such as trying clothes and displaying detailed information, which develops consumers’ trust in the seller as well as in the product and ultimately help in making a purchase decision for the particular product. This interaction on live streaming enhances the consumers’ shopping experience and reduces consumers’ uncertainty, increasing the seller’s trust level (Hajli, 2015; Haimson and Tang, 2017). Moreover, Li et al. (2018) suggested that two-way synchronized communication developed social presence and interaction between buyer and seller, and comments by the other viewers reduce uncertainty and increase trust in the seller. Because they thought the person on the other side was real, social and identified by the shoppers (Wongkitrungrueng and Assarut, 2018). Based on this discussion, it can be hypothesized that.

*H5*: Interactivity is positively and significantly associated with trust.

### 2.9. Trust

In the context of social commerce, a belief state that submits to the vulnerability caused by another party’s activities without keeping an eye on or exerting control over the other party is commonly referred to as trust (Al-Adwan and Kokash, 2019). “Trust” refers to the perception that the other party in a social trade will act morally and in a way that is acceptable for society and will not be opportunistic (Gefen et al., 2003; Yahia et al., 2018). Customer trust differs in different industries, and building trust is crucial for any business application (Bugshan and Attar, 2020). Building trust is critical to online commerce because customers can determine whether to buy things by accepting information about the products offered by vendors and members’ comments (Wongkitrungrueng and Assarut, 2018).

Customers can communicate with sellers and community members through s-commerce, which also aids community building. These individuals frequently talk about their purchasing experiences and particular products they find tremendous or awful, which helps them reduce online shopping risk. Live streaming has made a real-time experiential connection in chat rooms more popular than based-text human-computer contact. Customers can connect with streamers and community members while viewing and visualizing actual products through live-streaming commerce (i.e., visualization, authenticity, and interaction in real-time), which promotes buyers’ trust in both the sellers and the products (Zhang et al., 2022).

In this respect, buyers’ and sellers’ interactions are facilitated by trust in e-commerce, particularly live-streaming commerce (Kim et al., 2008). According to Chiu et al. (2009), trust can result in positive feelings toward the online merchant and thus improve the likelihood that a customer will return to and make a purchase. It significantly impacts consumers’ purchasing decisions relating to online retailers (Chang and Chen, 2008). The relationship between customer trust and engagement in the live-streaming context was examined by Wongkitrungrueng and Assarut (2018), who found a positive association.

*H6*: Trust is positively and significantly associated with intention.

## 3. Research method

A survey method was adopted in the current study. The questionnaire was developed on the bases of previously developed instruments. The instrument was distributed into two sections. The first section includes demographic information, while the second contains items of eight variables. Metavoicing affordance, shopping guidance affordance, visibility affordance and trading affordance were adapted from Dong and Wang (2018); interactivity was adapted from Ma et al. (2022), while trust in the seller and confidence in products were adapted from the research of Wongkitrungrueng and Assarut (2018). The only dependent variable, i.e., purchase intention, was adopted by Sun et al. (2019) (see Appendix A). The current study used a 7-point Likert scale to measure each item (1 = strongly disagree to 7 = strongly agree). As the items were initially in English, we used the forward-backwards translated method to translate items into Chinese (Sun et al., 2019; Ma et al., 2022). For this purpose, some field experts translated items from English to Chinese. Then five researchers translated the Chinese version into English back. Then other four University researchers compared the Chinese version, and there were no significant differences. After the content analysis, the pilot study was conducted. The questionnaire was distributed among 70 university students who have used live-streaming commerce. The reliability and validity tests were well above the threshold value.

The current study focused on social commerce platforms with live-streaming options, such as Taobao.com, Mogujie.com, and JD.com. These live-streaming platforms have the most prominent e-commerce consumers in China. The current study used multiple live streaming platforms rather than one to find consumers’ purchase intention from live streaming compared to a specific platform. Because consumers’ experiences can be different on different platforms, the results will represent the customers’ actual experiences. The questionnaires were distributed on the Wenjuanxing website,^1^ on which more than one million respondents answer daily and considered one of the largest data collection websites (Lin et al., 2018; Sun et al., 2019). The sample service was also used by Wenjuanxing, which enabled us to select consumers and help to remove invalid responses randomly. A pre-screening question was also asked from respondents to prioritize only those users who have experienced live streaming at least once in their lifespan. The questionnaire was only shared with the Respondents having live streaming experience. Then these respondents were asked to answer questions based on their experiences with the live-streaming shopping platform(s) in total, 434 questionnaires were valid and used for the analysis. According to Churchill and Iacobucci (2006), the sample size should be between 200 and 500 for behavioral studies. So our study’s sample size is appropriate for the analysis. The demographic profile of the users is given in Table 1.

**Table 1:** Demographic profile.

Item		Frequency	Percentage
Gender	Male	192	44.24
	Female	242	55.76
Age	20 or less than 20	23	5.29
	21–25	123	28.34
	26–30	146	33.64
	31–35	88	20.28
	36–40	32	7.37
	More than 41	22	5.07
Education level	High school or below	38	8.76
	Junior college or university degree	279	64.29
	Postgraduate	102	23.50
	Other	15	3.45
Monthly income (RMB)	Less than 2000	35	8.06
	2,001–3,500	47	10.83
	3,501–5,000	74	17.05
	5,001–6,500	61	14.05
	6,501–8,000	96	22.13
	More than 8,000	121	27.88
Profession	Student	121	27.88
	Corporate	77	17.74
	Government	170	38.18
	Freelancer or Business	56	12.90
	Others	10	2.30
Live streaming frequency (per month)	1–3	278	64.06
	4–6	107	24.65
	7–9	49	11.29
Live streaming platform usage	Taobao	396	91.24
	JingDong	253	58.29
	MoGujie	211	48.61

## 4. Data analysis and results

**Figure 1:**
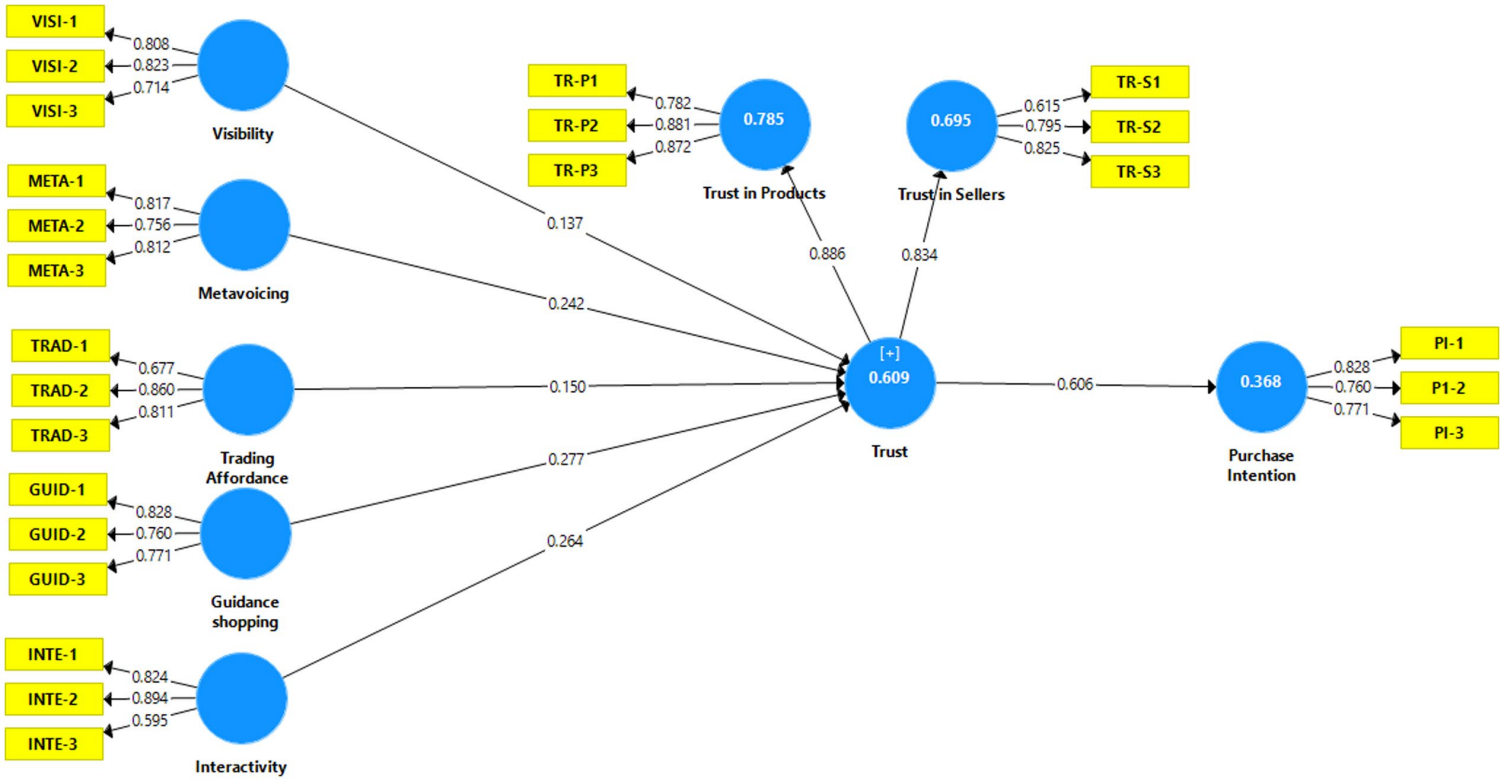
Measurement model.

Structural equation modeling (SEM) was used for data analysis in the current study. Because SEM is second generation analysis technique which has many advantages over first-generation multivariate analysis techniques, such as convenience, efficiency and accuracy (Ali et al., 2021a) and has become very popular in business studies due to its effectiveness (Cheah et al., 2019). There are two types of SEM, i.e., Partial least Square-SEM or variance-based SEM (PLS-SEM) and covariance-based SEM (CB-SEM) (Chin and Newsted, 1999; Ali et al., 2019). It is crucial to select appropriate data analysis techniques because inappropriate methods can lead to misleading results (Ramayah et al., 2010). So the current studies have adopted PLS-SEM because it is more suitable to analyze complex models and advanced statistical analysis (Hair et al., 2011). There are two main reasons to select PLS-SEM in the current study. First, in social sciences studies, data tend to have normality issues (Osborne, 2010). In this regard, PLS-SEM covered the normality issue and adequately handled the non-normal data (Ali et al., 2021b). Second, PLS-SEM improves the accuracy of the empirical results and is beneficial in exploratory studies compared to other behavioral studies techniques (Ramli et al., 2018). So, the current research is utilizing Smart PLS 3.0 for hypotheses testing. PLS-SEM is a two-step process which includes measurement model assessment and structural model assessment. Measurement model analysis covers reliability and validity tests, while structure model analysis covers the testing significance of hypotheses (Figure 1).

### 4.1. Measurement model analysis

**Figure 2:**
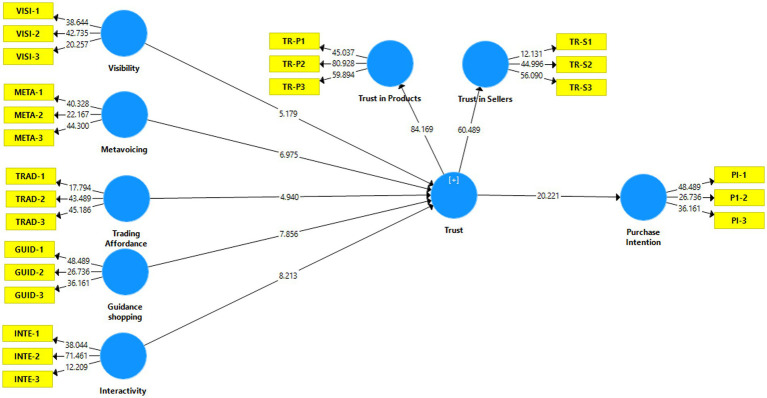
Structural model.

The measurement model analyzes internal consistency reliability, construct reliability, convergent validity and discriminant validity of the model. To find internal consistency reliability, composite reliability was used in the current study. Table 2 indicate that CR range from 0.792 (trust in seller) to 0.843 (trust), which indicates that CR values exceed the threshold value of 0.7 (Fornell and Larcker, 1981; Anderson and Gerbing, 1988). Convergent validity includes average variance extracted (AVE), which ranges from 0.564 (Trust in seller) to 0.716 (Trust in products), which also fulfills the recommended value (see Table 2; Fornell and Larcker, 1981). Outer loading was used to test the convergent validity. The results indicated that all values are well above the recommended value of 0.5 (see Table 2 and Figure 2; Hair et al., 2014).

**Table 2:** Factor loading, reliability analysis and convergent validity.

First-order constructs	Second-order constructs	Items	Loading	AVE	CR
Guidance shopping		GUID-1	0.828	0.619	0.830
		GUID-2	0.760		
		GUID-3	0.771		
Visibility		VISI-1	0.808	0.614	0.826
		VISI-2	0.823		
		VISI-3	0.714		
Metavoicing		META-1	0.817	0.632	0.838
		META-2	0.756		
		META-2	0.812		
Trade affordance		TRAD-1	0.677	0.618	0.828
		TRAD-2	0.860		
		TRAD-3	0.811		
Interactivity		INTE-1	0.824	0.611	0.821
		INTE-2	0.894		
		INTE-3	0.595		
Trust in products		TR-P1	0.782	0.716	0.883
		TR-P2	0.881		
		TR-P3	0.872		
Trust in seller		TR-S1	0.615	0.564	0.792
		TR-S2	0.795		
		TR-S3	0.825		
	Trust	TR-P	0.886	0.740	0.851
		TR-S	0.834		
Purchase intention		PI-1	0.828	0.619	0.830
		PI-2	0.760		
		PI-3	0.771		

According to Kline (2015), if the value is higher than 0.85, there is an issue of discriminant validity. To find discriminant validity, the current study adopted Heterotrait–Monotrait (HTMT) ratio rather than Fornell–Larcker (Hair et al., 2014). Table 3 shows no discriminant validity issue, as all values are less than the suggested value of 0.85.

**Table 3:** Discriminant validity-HTMT.

	Guidance shopping	Interactivity	Metavoicing	Purchase intention	Trading affordance	Trust in products	Trust in sellers	Visibility
Guidance shopping								
Interactivity	0.434							
Metavoicing	0.694	0.680						
Purchase intention	0.440	0.434	0.694					
Trading affordance	0.509	0.465	0.534	0.509				
Trust in products	0.610	0.626	0.612	0.610	0.543			
Trust in sellers	0.786	0.750	0.791	0.786	0.652	0.683		
Visibility	0.681	0.400	0.557	0.681	0.586	0.621	0.620	

### 4.2. Structural model analysis

#### 4.2.1. Testing model fitness

Before proceeding to test hypotheses, the current study conducted a model fitness test using two parameters: the Standardized Root Mean Square Residual (SRMR) and the Normed Fit Index (NFI). The SRMR value is 0.063, less than the threshold value of 0.08 (Hu and Bentler, 1998). For the model fit, the NFI value should be greater than 0.90, confirmed in the current study having a value of 0.952 (Bentler and Bonett, 1980).

#### 4.2.2. Hypotheses testing

After completing the measurement model assessment, the structural model was analyzed. The structural model covers five tests, i.e., t values, path coefficients (β values), effect size (f2), predictive relevance (Q2) and coefficient of determination (R2). A bootstrapping method (5,000 resample) was used to calculate β values. Table 4 indicates the empirical results. The results indicated that guidance shopping affordance (*β* = 0.277, *t* = 7.856 > 1.64), metavoicing affordance (*β* = 0.242, *t* = 6.975 > 1.64), visibility affordance (*β* = 0.137, *t* = 5.179 > 1.64), trading affordance (*β* = 0.150, *t* = 4.940 > 1.64), and interactivity affordance (*β* = 0.264, *t* = 8.213 > 1.64) has positive association with trust. Moreover, trust (*β* = 0.606, *t* = 7.856 > 20.221) positively associates with live-streaming purchase intention. The results showed that H1-H6 are supported, i.e., guidance shopping affordance, metavoicing affordance, visibility affordance, trading affordance and interactivity affordance impact on consumers trust, which is a second-order variable developed by the trust on seller and trust on buyer. Trust ultimately impact the consumer purchase intention from live-streaming commerce. The R2 of purchase intention is 0.368, indicating that the developed model explained a 36.8% variation in purchase intention. The value of R2 is moderate as per Cohen (1988) suggestion, as 0.18 is considered weak, 0.25 moderate and 0.40 substantial. Effect size depicts the impact of exogenous variables on the endogenous variable (Hair et al., 2014; Akbar et al., 2019) according to Cohen (1988), the effect size is considered weak, moderate and strong if the value of F2 is under 0.02, 0.15, 0.35.

**Table 4:** Structural model results.

Hypothesis	Relationships	Path coefficients	Std Error	*T*-value	*P*-value	Supported	F^2^	Q^2^	R^2^
H1	GUID- > Trust	0.277	0.035	7.856	0.00	Yes	0.27	0.211	0.368
H2	META- > Trust	0.242	0.035	6.975	0.00	Yes	0.18		
H3	VISI- > Trust	0.137	0.026	5.179	0.00	Yes	0.10		
H4	TRAD- > Trust	0.150	0.030	4.940	0.00	Yes	0.13		
H5	INTE- > Trust	0.264	0.032	8.213	0.00	Yes	0.23		
H6	Trust- > PI	0.606	0.030	20.221	0.00	Yes	0.32		

## 5. Discussion and conclusion

### 5.1. Discussion

The primary objective of this research is to analyze how live-streaming shopping affects consumers’ intentions to make purchases through social commerce platforms. To do this, we developed a research model that emphasizes the affordability of IT and consumer trust. All of our hypotheses were confirmed by the findings of the empirical investigation, which demonstrates that the model is suitable for research on live streaming commerce. Our research shows that trust in both the products and the sellers during live-streaming shopping positively correlates with the customer’s intent to purchase. This trust is directly influenced by IT affordances (including visibility, metavoicing, trading affordance, guidance shopping and interactivity) which ultimately leads toward the consumers’ purchase intention. The result is in line with previous researchers, e.g., (Sun et al., 2019) found that consumers’ purchase intention is influenced by IT affordance (guidance shopping, metavoicing and visibility).

Similarly, the current study also confirmed the results of the (Dong and Wang, 2018) who found the positive influence of the interactivity and trading affordance on the consumers intentions. The results align with previous research (Wongkitrungrueng and Assarut, 2018). The possible reason for the results is that live streaming helps consumers to reduce the risk of shopping for a product by watching original products, interacting with sellers and others, getting suggestions from the sellers, and exchanging products and related transaction information. The possible reason for the result can be that when consumers watch their product live, it increases their confidence in the product and the sellers and reduces the risk of fraud. So they ultimately intend to buy those products.

### 5.2. Theoretical implication

Our investigation contributes to the existing body of literature by presenting three essential insights about live-streaming shopping. First, our research clarifies how the IT affordance and customer trust perspectives on live streaming affect consumer purchase intention. Many facets of conventional forms of social commerce have been altered due to live streaming, but prior research has not studied the effects of these changes. After reviewing previous research, we investigated consumers’ points of view from the IT affordance perspective (Dong and Wang, 2018; Sun et al., 2019; Ma et al., 2022). Our findings indicate that live-streaming shopping affordances, such as visibility affordance, metavoicing affordance, and guidance shopping affordance, may affect consumer buy intention *via* live-streaming shopping customer trust. Additionally, we define live-streaming shopping trust as the mechanism through which live-streaming affordance influences consumer buying intention. As a result, this research lays the conceptual framework for further research on live-streaming retail environments.

Additionally, our study predominantly employs the affordance lens, an emerging research viewpoint for live-streaming shopping. Previous research on social commerce and live-streaming shopping has independently investigated the elements of social commerce and how buyers perceive such aspects. Affordance is a notion that may assist researchers in taking into consideration not just features but also how buyers perceive them. Third, we develop the concept of consumer trust (trust in product and trust in sellers) in live streaming commerce and demonstrate that it offers a way through which IT affordance may affect customers’ intentions to make a purchase.

### 5.3. Managerial implications

This research also provides actionable guidelines for marketers and live-streaming commerce practitioners. First, streamers and managers should value social interaction and IT affordance to enhance customers’ trust. In addition to providing a theoretical framework, this study offers practical recommendations for live-streaming business marketing. Several valuable recommendations for live stream designers and social commerce vendors are derived from these findings. Our research reveals that the IT affordances of live streaming shopping—such as visibility, metavoicing, guidance shopping, interactivity and trading —each independently affect consumers’ intention to purchase the products. Guidance shopping has the most significant influence on trust Streamers need to provide an individual experience for their customers. Comparable to automated product suggestions, it can “take as input individual consumers’ product-related interests or preferences, either explicitly or implicitly, and subsequently provide recommendations for products that match the consumer’s expressed interests or preferences.” In addition, interactivity has a more significant influence on trust. Streamers should intensify and enrich the communication between the two sides.

Moreover, metavoicing also significantly affects consumer trust. Streamers could better serve customer demands for accessing pertinent information about goods and services. Streamers and managers should prioritize IT affordances to increase consumers’ trust. Streamers should be quick to answer inquiries from viewers. They should use extensive visual means to showcase items for viewers from all angles, giving them a comprehensive and immersive product experience Customers’ skepticism about the products may be addressed, and their trust in the streamer can be strengthened in this manner. At the same time, streamers and managers should think about giving clients more sophisticated control choices (such as zooming in and out, blocking the voice box, etc.) to provide them with a better experience.

### 5.4. Limitations and future research

The scope and depth of our investigation are limited in several ways. First, we collect data about consumer buying intentions. Although buying intention may predict purchase behavior, actual purchase data are more reliable in determining such behavior. If the situation allows, future research should employ transactional data to examine consumer purchasing patterns during live streaming.

Second, the participants in Chinese live-streaming commerce made up our sample; as a result, the conclusions drawn from this study may not have as much relevance to the general population. Live streaming commerce in China is more established than in other countries, and most research done in this field in the past concentrated on the Chinese environment. To assess whether the present study model has excellent external validity, future research may consider live-streaming consumers in other countries. Along these lines, future studies may include cultural differences in the model. This is because the samples that are now available are mainly oriented toward Chinese culture. An examination of Chinese culture with western culture has the potential to provide more excellent light on the topic.

## Data availability statement

The raw data supporting the conclusions of this article will be made available by the authors, without undue reservation.

## Author contributions

LZ and MC participated in the manuscript’s conceptualization and methodology. AZ participated in writing, and revisions, made a substantial and intellectual contribution to the work. MC did the data collection. All the authors approved it for publication.

## Conflict of interest

The authors declare that the research was conducted in the absence of any commercial or financial relationships that could be construed as a potential conflict of interest.

## Publisher’s note

All claims expressed in this article are solely those of the authors and do not necessarily represent those of their affiliated organizations, or those of the publisher, the editors and the reviewers. Any product that may be evaluated in this article, or claim that may be made by its manufacturer, is not guaranteed or endorsed by the publisher.

## Footnotes

^1^ https://www.wjx.cn/

## Appendix A

**Guidance shopping**

GUD1: Streamers on live streaming shopping can provide information on all alternative products I intend to buy.

GUD2: Streamers on live streaming shopping can help me establish my product needs without restrictions.

GUD3: Streamers on live streaming shopping can help me identify which product attributes best fit my needs.

**Metavoicing**

META1: Live streaming shopping allows me to comment on products.

META2: Live streaming shopping allows me to react to streamers’ feedback on products.

META3: Live streaming shopping allows me to share streamers’ opinions about products.

**Visibility**

VISI1: Live streaming shopping provides me with detailed pictures and videos of the products.

VISI2: Live streaming shopping makes the product attributes visible to me.

VISI3: Live streaming shopping makes information about how to use products visible to me.

**Trading**

TRA1: The live streamers me multiple payment options to complete a purchase.

TRA2: The live streamers help me to finish a transaction in an effective way.

TRA3: The live streamers enable me to complete a transaction smoothly.

**Interactivity**

INTE1: The live streamers were very happy to communicate with viewers.

INTE2: The live streamers actively responded to viewers’ questions.

INTE3: The live streamers answered viewers’ questions and requests in time.

**Trust in seller**

TR-S1: I believe in the information the social media seller provides through live streaming.

TR-S2: I can trust social media sellers that use live streaming.

TR-S3: I believe that social media sellers who use live streaming are trustworthy.

**Trust in product**

TR-P1: I think the products I order from Live streaming will be as I imagined.

TR-P2: I believe that I will be able to use products like those demonstrated on Live streaming.

TR-P3: I trust that the products I receive will be the same as those shown on Live streaming.

**Purchase intention**

PI1: I will consider live streaming shopping as my first shopping choice.

PI2: I intend to purchase products or services through live streaming shopping.

PI3: I expect that I will purchase products or services through live streaming shopping.
